# Non-patient-related SARS-CoV-2 exposure from colleagues and household members poses the highest infection risk for hospital employees in a German university hospital: follow-up of the prospective Co-HCW seroprevalence study

**DOI:** 10.1007/s15010-023-01995-z

**Published:** 2023-02-15

**Authors:** Christina Bahrs, Sebastian Weis, Miriam Kesselmeier, Juliane Ankert, Stefan Hagel, Stephanie Beier, Jens Maschmann, Andreas Stallmach, Andrea Steiner, Michael Bauer, Wilhelm Behringer, Michael Baier, Cora Richert, Florian Zepf, Martin Walter, André Scherag, Michael Kiehntopf, Bettina Löffler, Mathias W. Pletz

**Affiliations:** 1 Institute for Infectious Diseases and Infection Control, Jena University Hospital/Friedrich-Schiller-University, Am Klinikum 1, 07747 Jena, Germany; 2 Department of Medicine I, Division of Infectious Diseases and Tropical Medicine, Medical University of Vienna, Vienna, Austria; 3 Leibniz Institute for Natural Product Research and Infection Biology-Hans Knöll Institute (HKI), Jena, Germany; 4 Institute of Medical Statistics, Computer and Data Sciences, Jena University Hospital/Friedrich-Schiller-University, Jena, Germany; 5 Medical Executive Board, Würzburg University Hospital, Würzburg, Germany; 6 Department of Internal Medicine IV (Gastroenterology, Hepatology and Infectious Diseases), Jena University Hospital/Friedrich-Schiller-University, Jena, Germany; 7 Department for Occupational Health, Jena University Hospital/Friedrich-Schiller-University, Jena, Germany; 8 Department of Anesthesiology and Intensive Care Therapy, Jena University Hospital/Friedrich-Schiller-University, Jena, Germany; 9 Department of Emergency Medicine, Medical University of Vienna, Vienna, Austria; 10 Institute of Medical Microbiology, Jena University Hospital/Friedrich-Schiller-University, Jena, Germany; 11 Department of Clinical Chemistry and Laboratory Medicine, Jena University Hospital/Friedrich-Schiller-University, Jena, Germany; 12 Department of Child and Adolescent Psychiatry, Psychosomatic Medicine and Psychotherapy, Jena University Hospital/Friedrich-Schiller-University, Jena, Germany; 13 Department of Psychiatry and Psychotherapy, Jena University Hospital/Friedrich-Schiller-University, Jena, Germany

**Keywords:** SARS-CoV-2 infection, Seroepidemiological studies, Healthcare workers, Universal masking, Non-patient-related COVID-19 contact

## Abstract

**Purpose:**

The Co-HCW study is a prospective, longitudinal, single-center observational study that aims to assess the SARS-CoV-2 seroprevalence and infection status in staff members of Jena University Hospital (JUH) in Jena, Germany.

**Methods:**

This follow-up study covers the observation period from 19th May 2020 to 22nd June 2021. At each of the three voluntary study visits, participants filled out a questionnaire regarding their SARS-CoV-2 exposure and provided serum samples to detect specific SARS-CoV-2 antibodies. Participants who were tested positive for antibodies against nucleocapsid and/or spike protein without previous vaccination and/or reported a positive SARS-CoV-2 PCR test were regarded to have been infected with SARS-CoV-2. Multivariable logistic regression modeling was applied to identify potential risk factors for infected compared to non-infected participants.

**Results:**

Out of 660 participants that were included during the first study visit, 406 participants (61.5%) were eligible for the final analysis as their COVID-19 risk area (high-risk *n* = 76; intermediate-risk *n* = 198; low-risk *n* = 132) did not change during the study. Forty-four participants [10.8%, 95% confidence interval (95%CI) 8.0–14.3%] had evidence of a current or past SARS-CoV-2 infection detected by serology (*n* = 40) and/or PCR (*n* = 28). No association between SARS-CoV-2 infection and the COVID-19 risk group according to working place was detected. However, exposure to a SARS-CoV-2 positive household member [adjusted OR (AOR) 4.46, 95% CI 2.06–9.65] or colleague (AOR 2.30, 95%CI 1.10–4.79) was found to significantly increase the risk of a SARS-CoV-2 infection.

**Conclusion:**

Our results demonstrate that non-patient-related SARS-CoV-2 exposure posed the highest infection risk for hospital staff members of JUH.

**Supplementary Information:**

The online version contains supplementary material available at 10.1007/s15010-023-01995-z.

## Introduction

Healthcare workers (HCWs) across the world are at high risk of contracting coronavirus disease 2019 (COVID-19), caused by the severe acute respiratory syndrome coronavirus 2 (SARS-CoV-2) [1–3], due to their direct or indirect exposure to infectious material [3] while caring for patients suffering from the virus [4]. Transmission of SARS-CoV-2 is primarily through inhalation of, or inoculation with, infectious small liquid particles, ranging from larger respiratory droplets to smaller aerosols in the case of close contact [5]. Aerosol transmission in healthcare settings may occur in specific situations where HCWs perform aerosol-generating procedures without using adequate personal protective equipment (PPE) [5]. With the ongoing COVID-19 pandemic [6, 7], the safety of HCWs is of utmost relevance [1, 3, 5]. To reduce nosocomial transmissions, infection control measures such as the use of adequate PPE, hand hygiene, and physical separation are essential [5, 8]. Additionally, vaccination of patients and HCWs is an effective measure to reduce the risk of acquiring COVID-19 in healthcare settings.

The city of Jena, with a population of approximately 111,000 inhabitants, hosts the only university hospital in the central German state of Thuringia, Jena University Hospital (JUH). In March 2020, the local medical executive board implemented mandatory masking for all JUH staff, including HCWs and administration staff [9], to reduce nosocomial SARS-CoV-2 transmissions. Furthermore, all employees were prohibited from participating in business trips, conferences or trainings outside of JUH in person. In December 2020, the first SARS-CoV-2 vaccinations became available and were initially offered to HCWs at high risk. Since February 2021, SARS-CoV-2 vaccination has been offered to all hospital staff members. According to the department of occupational health of JUH, the vaccination rate in December 2021 was 85% (94% for physicians, 88% for nurses, and 85% for administration staff). Our previous study revealed a low SARS-CoV-2 point seroprevalence rate of 2.7% among hospital staff (inclusion of first participant: 19th May 2020, inclusion of last participant: 19th June 2020) [9]. We identified COVID-19 exposure at home as the main risk factor associated with SARS-CoV-2 point seroprevalence, prior to the availability of SARS-CoV-2 vaccination.

The primary objective of this follow-up study was to assess the SARS-CoV-2 seroprevalence and prevalence of SARS-CoV-2 infection among employees with (HCWs) and without patient contact (administration staff) of JUH over a period of 13 months (May 2020 to June 2021). Additionally, we sought to determine individual exposure risk factors, and to compare SARS-CoV-2 infection rates between hospital staff working at different COVID-19 risk areas according to working place.

## Methods

### Study design and setting

The Co-HCW study (SARS-CoV-2 seroprevalence and infection status in hospital staff members at JUH) is a prospective, longitudinal, single-centre observational cohort study conducted at JUH, a 1400-bed academic hospital in Germany. The first of three visits (05/2020) has already been published [9], and this current analysis covers the complete observation period of 11–13 months, from 19th May 2020 to 22nd June 2021. At our hospital, intensive SARS-CoV-2 screening was conducted to ensure the safety of our staff and patients. Details of the routine PCR screening are described below.

### Enrollment, risk area definitions and study measures

From 19th May to 19th June 2020, participants including hospital staff and administration staff were recruited for the study. All participants provided informed consent and met the inclusion and exclusion criteria outlined in the previously published results of the first study visit [9]. Three study visits were offered to all participants, with participation in each study visit being voluntary. The first study visit was performed at the time of inclusion, the second from 6th November to 26th November 2020, and the third study visit was performed from 26th April to 22nd June 2021. For the present analysis, only participants who completed the last study visit in 2021 and did not change the COVID-19 risk area according to their risk of contact with COVID-19 patients at work (low, intermediate, and high risk) during the study were considered. Briefly, the low-risk group consisted of administration staff who had no contact with patients. The intermediate-risk group included HCWs who had regular contact with patients, but typically did not treat those with confirmed or suspected SARS-CoV-2 infections. Lastly, the high-risk group was comprised of HCWs who worked in areas with confirmed COVID-19 patients, such as COVID-19 normal wards or ICUs, as well as areas that dealt with a high number of suspected COVID-19 cases, such as the emergency medicine and occupational health [9].

At each study visit, participants were required to fill out a questionnaire and provide blood samples which were sent to the Department of Clinical Chemistry and Laboratory Medicine of JUH and the Institute of Medical Microbiology of JUH for testing of specific SARS-CoV-2 antibodies by two different immunoassays (see below).

### Questionnaire

As previously described [9], the questionnaire included questions on demographics, profession, working area, individual exposure to confirmed COVID-19 cases, return from COVID-19 risk areas, results of previous polymerase chain reaction (PCR) or serology test for COVID-19, clinical symptoms, accidents with biological material, and compliance concerning use of PPE in HCWs when interacting with a confirmed COVID-19 patient. For the second and third visits, the questionnaire was updated to include questions about the use of public transport on the way to work, household size, travel to abroad, and participation at events with at least five persons. With the availability of SARS-CoV-2 vaccination since 27th December 2020, the questionnaire for the last visit was further extended to inquire about the number and type of SARS-CoV-2 vaccinations.

### PCR screening

All staff in high-risk areas (intensive care unit, intermediate care unit, emergency department, and COVID-19 regular ward) were routinely screened twice weekly for SARS CoV-2 via real-time (RT) PCR. To facilitate this, HCWs self-collected a nasopharyngeal sample. Additionally, if staff members experienced symptoms of infection and/or had contact with a SARS-CoV-2 infected person at work or at home, they were asked to have a nasopharyngeal sample collected by a HCW of the department of occupational health. Furthermore, in the event of nosocomial transmission detected by patient screening, a HCW of the department of occupational health took a nasopharyngeal sample from the staff of the respective ward on days 1 and 5. Detection of SARS CoV-2 was performed by RT-PCR amplification of SARS-CoV-2 E-gene [RNA-extraction: QIASymphony instrument, QIAsymphony DSP Virus/Pathogen MiniKit (Qiagen, Hilden, Germany), amplification/detection: LightCycler 480 II instrument (F. Hoffmann-La Roche AG, Basel, Switzerland), LightMix Modular Sarbecovirus E-gene kit (TIB MOLBIOL, Berlin, Germany)] and N/Nsp-gene (NeuMoDx SARS-CoV-2 Assay, NeuMoDx Molecular, Ann Arbor, USA) according to the manufacturers’ instructions [10].

### SARS-CoV-2 antibody testing

At each time point, specific SARS-CoV-2 antibodies were detected in serum samples using a commercially available chemiluminescence-based immunoassay (CLIA) Elecsys Anti-SARS-CoV-2 (Roche, Basel, Switzerland) that utilizes a recombinant nucleocapsid protein as a capture antigen. At the first and second visits, an enzyme-linked immunosorbent assay, EDI Novel Coronavirus SARS-CoV-2 IgG ELISA (Epitope Diagnostics Inc., San Diego, USA, antigen: recombinant nucleocapsid protein), was performed as a second method. At the third visit, spike-protein specific IgG antibodies were identified using the CLIA system LIAISON^®^ SARS CoV-2 S1/S2 IgG (DiaSorin, Saluggia, Italy). All serological tests were carried out according to the manufacturers’ instructions, and the manufacturers reported high sensitivities and specificities for all tests (≥ 97%).

Participants who were tested positive for antibodies against nucleocapsid and/or spike protein without previous vaccination and/or reported a positive SARS-CoV-2 PCR test were regarded to have been infected with SARS-CoV-2.

### Outcomes 

The primary outcome of this follow-up study was to assess the SARS-CoV-2 infection rates using SARS-CoV-2 antibody detecting immunoassays and reported positive SARS-CoV-2 PCR test results. Secondary outcomes were (i) determining the prevalence of SARS-CoV-2 infection in participants stratified by their risk of COVID-19 exposure during work (low, medium and high risk), and (ii) identifying potential risk factors for detected SARS-CoV-2 infection, such as compliance of HCWs in the event of an individual reported contact with a confirmed COVID-19 positive patient.

### Statistical analysis

Characteristics of participants were summarized overall and stratified by test result as absolute and relative frequencies or as median values with first and third quartiles (Q1, Q3). Evidence of a SARS-CoV-2 infection in hospital staff within the observation period was described with absolute and relative frequencies together with 95% Clopper-Pearson confidence intervals (CIs). To compare SARS-CoV-2 infection rates between participants working at different COVID-19 risk areas, and to identify potential risk factors for infected compared to non-infected participants, we applied uni- and multivariable logistic regression modeling with the SARS-CoV-2 infection as dependent variable and the investigated factors as independent variables. In the multivariable models, we adjusted for age and gender. For place of exposure, we considered two additional multivariable models. In the first additional model, we included all places that were assessed as independent variables to adjust each investigated place for the respective other places. In the second additional model, we adjusted this model for age and gender. We provided (adjusted) odds ratios (OR) together with 95% CI and *p* values. Additionally, we performed a subgroup analysis (separated for males and females) of all potential risk factors.

We applied a two-sided significance level of 0.05 and did not correct for multiple testing as all analyses were considered exploratory. The main analyses were done with R (version 4.0.3), and parts were complemented by SPSS Statistics version 28.0 for Windows (IBM Corp., Armonk, NY, USA).

## Results

### Characteristics of the study population

Out of the 660 participants analyzed during the first study visit, 406 hospital staff members (61.5%) also returned for the third and last study visit, remaining in the same COVID-19 risk area for the 13 month period. Of these 406 participants, 91 (22.4%) were male and 315 (77.6%) were female, with a median age of 41.0 (Q1–Q3 34.0–49.8) years. The most common professions included administration staff (*n* = 132, 32.5%), followed by nurses (*n* = 125, 30.8%), physicians (*n* = 66, 16.3%), reception staff (*n* = 12, 3.0%), nursing assistants (*n* = 10, 2.5%), psychologists (*n* = 10, 2.5%), ergo therapists (*n* = 10, 2.5%), and medical assistants (*n* = 9, 2.2%). Two-hundred twenty-four participants (55.2%) reported direct contact with a confirmed COVID-19 case, while 182 participants (44.8%) were not aware of any COVID-19 exposure. Of the 224 staff members with reported COVID-19 exposure, 151 participants (67.4%) had direct contact with a SARS-CoV-2 positive patient, and 60 participants (26.8%) had exposure to a SARS-CoV-2 positive colleague. Additionally, 43 participants (19.2%) reported close contact to a positive household member, 20 participants (8.9%) reported exposure to friends, 2 participants (0.9%) reported exposure during shopping, and 1 participant (0.4%) reported exposure on holiday. Further details on the participants are provided in Table 1. Three hundred and seven participants (75.6%) reported having received a SARS-CoV-2 vaccination prior to their last study visit. Of these, 177 participants (43.6%) had received two vaccinations: 160 had a homologous vaccination with a COVID-19 messenger RNA (mRNA) vaccine, 7 had a homologous vaccination with the vector-based vaccine ChAdOx1-S, and 10 had a heterologous vaccination with the vector-based vaccine followed by an mRNA vaccine. The remaining 130 participants (32.0%) had received one vaccination: 16 had an mRNA vaccine, and 114 had a vector-based vaccine.

**Table 1:** Potential risk factors for a current or past SARS-CoV-2 infection (detected by serology and/or PCR) among hospital staff members

Variable	Overall (*N* = 406)	Detected infection		Univariable analysis		Multivariable analysis	
		Yes (*N* = 44)	No (*N* = 362)	OR (95% CI)	*p* value	Adjusted OR (95% CI)	*p* value
Age, in years	41.0 (34.0, 49.8)	43.0 (32.0, 51.0)	41.0 (34.0, 49.0)	1.00 (0.97, 1.03)	0.969	1.00 (0.97, 1.03)	0.962
Male gender	91 (22.4%)	11 (25.0%)	80 (22.1%)	1.17 (0.57, 2.43)	0.663	1.18 (0.57, 2.43)	0.663
Profession							
Physician	66 (16.3%)	2 (4.5%)	64 (17.7%)	Ref	0.107	Ref	0.108
Nurse	125 (30%)	18 (40.9%)	107 (29.6%)	5.38 (1.21, 23.97)	0.027	5.57 (1.24, 25.12)	0.025
Reception staff	12 (3.0%)	1 (2.3%)	11 (3.0%)	2.91 (0.24, 34.89)	0.400	3.05 (0.25, 37.65)	0.348
Administration staff	132 (32.5%)	17 (38.6%)	115 (31.8%)	4.73 (1.06, 21.13)	0.042	4.92 (1.07, 22.64)	0.041
Other profession	71 (17.5%)	6 (13.6%)	65 (18.0%)	–	–	–	–
COVID-19 risk group according to working place							
High-risk	76 (18.7%)	7 (15.9%)	69 (19.1%)	Ref	0.643	Ref	0.644
Intermediate-risk	198 (48.8%)	20 (45.5%)	178 (49.2%)	1.11 (0.45, 2.74)	0.825	1.15 (0.46, 2.89)	0.763
Low-risk	132 (32.5%)	17 (38.6%)	115 (31.8%)	1.46 (0.58, 3.69)	0.427	1.52 (0.58, 3.98)	0.397
Reported COVID-19 exposure	224 (55.2%)	38 (86.4%)	186 (51.4%)	5.99 (2.47, 14.53)	< 0.001	7.19 (2.86, 18.11)	< 0.001
Among them: place of reported exposure							
Household member	43 (19.2%)	16 (42.1%)	27 (14.5%)	4.28 (2.00, 9.18)	< 0.001	4.46 (2.06, 9.65)	< 0.001
Friend	20 (8.9%)	2 (5.3%)	18 (9.7%)	0.52 (0.12, 2.33)	0.392	0.52 (0.11, 2.35)	0.394
Colleague	60 (26.8%)	16 (42.1%)	44 (23.7%)	2.35 (1.13, 4.86)	0.022	2.30 (1.10, 4.79)	0.026
Patient	151 (67.4%)	18 (47.4%)	133 (71.5%)	0.36 (0.18, 0.73)	0.005	0.36 (0.18, 0.75)	0.007
Other	3 (1.3%)	1 (2.6%)	2 (1.1%)	2.49 (0.22, 28.14)	0.462	2.60 (0.22, 30.41)	0.446
Accident with biological material	8 (2.0%)	2 (4.5%)	6 (1.7%)	2.83 (0.55, 14.45)	0.212	2.77 (0.54, 14.23)	0.222
Compliance to wear PPE^a^	133 (92.4%)	15 (88.2%)	118 (92.9%)	0.57 (0.11, 2.90)	0.500	0.58 (0.11, 2.94)	0.507
Use of public transport	36 (8.9%)	6 (13.6%)	30 (8.3%)	1.75 (0.68, 4.47)	0.244	1.77 (0.69, 4.54)	0.235
Household size							
Number of members	2.0 (2.0, 4.0)	2.5 (2.0, 4.0)	2.0 (2.0, 4.0)	0.99 (0.77, 1.27)	0.924	0.99 (0.77, 1.27)	0.918
> 1 member	319 (78.6%)	34 (77.3%)	285 (78.7%)	0.92 (0.43, 1.94)	0.824	0.92 (0.44, 1.95)	0.835
Returning from risk area	79 (19.5%)	10 (22.7%)	69 (19.1%)	1.25 (0.59, 2.65)	0.562	1.25 (0.59, 2.65)	0.562
Travel to abroad	99 (24.4%)	12 (27.3%)	87 (24.0%)	1.19 (0.59, 2.40)	0.637	1.20 (0.59, 2.44)	0.614
Participation at an event with ≥ 5 persons	197 (48.5%)	24 (54.5%)	173 (47.8%)	1.31 (0.70, 2.46)	0.398	1.32 (0.70, 2.51)	0.389

Overall and by infection detection stratified distribution of potential risk factors as well as results from uni- and multivariable logistic regression modeling are provided. Distributions are summarized as absolute and relative frequencies or as median together with the first and third quartile. All multivariable models were adjusted for age and sex. The complete models are provided in Supplemental Table 1. Further subgroup analysis (separated for males and females) of all potential risk factors are provided in Supplemental Table 2 and Supplemental Table 3

– Excluded from model, *CI* confidence interval, *N* number of, *OR* odds ratio, *PCR* polymerase chain reaction, *PPE* personal protective equipment, *ref* reference

^a^Information is missing for 262 participants who did not care for COVID-19 patients

### Seroprevalence and prevalence of SARS-CoV-2 infection

At the last study visit, 78.3% (295 vaccinated and 23 unvaccinated) of the 406 participants were tested seropositive by the Liaison test, while 21.7% (12 vaccinated and 76 unvaccinated) remained seronegative. Over the course of the 13 months observational period, 44 of 406 participants (10.8%, 95% CI 8.0–14.3%) had evidence of a SARS-CoV-2 infection detected by serology and/or PCR. As shown in Table 2, of these 44 participants, 40 participants (90.9%) had at least one positive SARS-CoV-2 IgG antibody test compatible with current or past infection (positive Roche test *n* = 30; positive EDI ELISA *n* = 13; positive Liaison test despite missing vaccination *n* = 26), and 28 participants (63.3%) reported at least one positive PCR test result. According to the self-reported symptoms, nine of the 44 infected participants (20.5%) had an asymptomatic SARS-CoV-2 infection, two (4.5%) had very mild symptoms, eight (18.2%) had mild symptoms, 14 (31.8%) had moderate symptoms, and eleven (25%) had severe symptoms.

**Table 2:** Evidence for a detected COVID-19 infection (PCR and/or antibody test result) among all hospital staff members, stratified according to their COVID-19 risk group based on their working place

Evidence	Overall	Risk group		
		High	Intermediate	Low
Evidence for COVID-19 among them	44 out of 406 participants (10.8%, 8.0% to 14.3%)	7 out of 76 participants (9.2%, 3.8% to 18.1%)	20 out of 198 participants (10.1%, 6.3% to 15.2%)	17 out of 132 participants (12.9%, 7.7% to 19.8%)
Evidence through PCR test				
Among all participants	28 (63.6%, 47.8% to 77.6%)	4 (57.1%, 18.4% to 90.1%)	16 (80.0%, 56.3% to 94.3%)	8 (47.1%, 23.0% to 72.2%)
Among participants with PCR test^a^	28 (71.8%, 55.1% to 85.0%)	4 (66.7%, 22.3% to 95.7%)	16 (80.0%, 56.3% to 94.3%)	8 (61.5%, 31.6% to 86.1%)
Evidence through antibody test	40 (90.9%, 78.3% to 97.5%)	5 (71.4%, 29.0% to 96.3%)	19 (95.0%, 75.1% to 99.9%)	16 (94.1%, 71.3% to 99.9%)

Values show number of participants (percentage, 95% confidence interval)

*PCR* polymerase chain reaction

^a^ PCR test results were reported in 39 of the 44 participants with a detected COVID-19 infection (by PCR and/or serology)

**Fig. 1:**
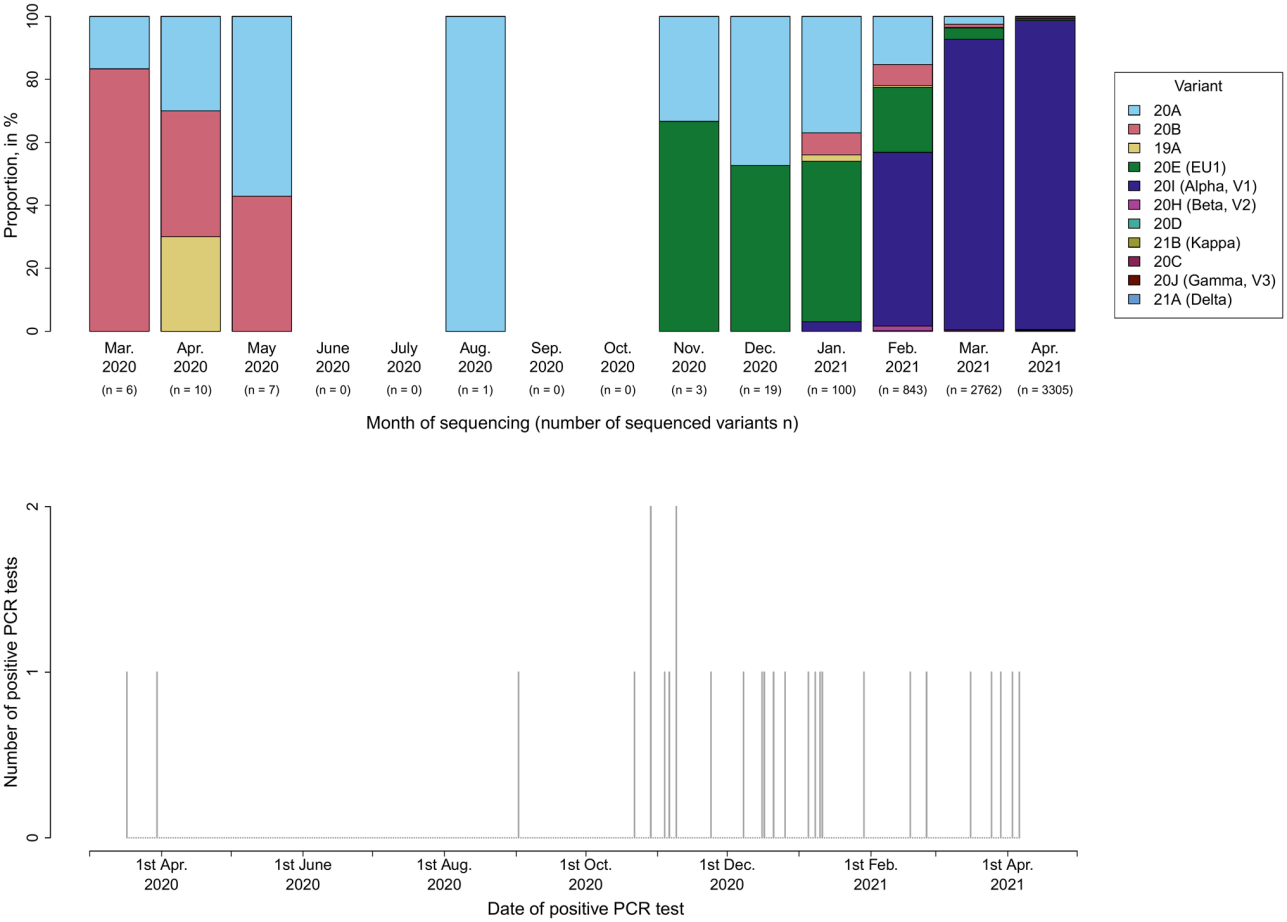
Distribution of SARS-CoV-2 variants from the Thuringian surveillance samples (upper panel) and number and time of reported positive PCR test results among hospital staff members (lower panel) during the period 1st March 2020 to 30rd April 2021. Variants sequenced by the Institute for Infectious Diseases and Infection Control (JUH) are shown. Concerning the data of the SARS-CoV-2 variants, we refer to https://charts.mongodb.com/charts-routine-sequencing-sars-c-amykg/public/dashboards/e9453286-1dce-4202-9423-a8459e3962f8. Underlying data were last assessed on 7th March 2022. Abbreviations: *JUH* Jena University Hospital, *PCR* polymerase chain reaction

As shown in Fig. 1, the majority of PCR test results (25/28, 89.2%) were positive during the last 6 months of the study. SARS-CoV-2 variants of concern (VOCs) Alpha, Beta, Gamma and Delta did not emerge in Thuringian surveillance samples until 2021 (Alpha variant since January 2021, Beta variant since February 2021, Gamma and Delta variants since April 2021). The molecular surveillance and the timeline for VOCs in the State of Thuringia can be found at https://charts.mongodb.com/charts-routine-sequencing-sars-c-amykg/public/dashboards/e9453286-1dce-4202-9423-a8459e3962f8.

Two PCR-positive, unvaccinated participants did not show any seroconversion. Breakthrough infections after vaccination, confirmed by a positive PCR test result, were reported in one participant 3 months after two vaccinations with the COVID-19 mRNA vaccine BNT162b2, in one participant six weeks after one vaccination with the vector-based COVID-19 vaccine ChAdOx1-S, and in one participant 4 months after only one vaccination with BNT162b2.

### Potential risk factors for evidence of a SARS-CoV-2 infection of staff members

As shown in Table 1, we did not find an association between a current or past SARS-CoV-2 infection (detected by serology and/or PCR) and demographics, household size, use of public transport to get to work, returning from an inner-German “COVID-19 risk area” as defined by national public health authorities according to the respective incidence, travel to abroad or participation at events with equal to or more than five persons, COVID-19 risk group according to working place, reported accident with biological material or compliance to wear PPE. However, professions associated with an increased risk of experiencing a SARS-CoV-2 infection compared to physicians included nurses (adjusted OR 5.57, 95% CI 1.24–25.12; *p* = 0.025) and administration staff (adjusted OR 4.92, 95% CI 1.07–22.64; *p* = 0.041). Additionally, a reported (occupational and private) COVID-19 exposure (adjusted OR 7.19, 95% CI 2.86–18.11; *p* < 0.001) and particularly close contact to a SARS-CoV-2 positive household member (adjusted OR 4.46, 95% CI 2.06–9.65; *p* < 0.001) and exposure to a SARS-CoV-2 positive colleague (adjusted OR 2.30, 95% CI 1.10–4.79; *p* = 0.026) significantly increased the risk of a SARS-CoV-2 infection among hospital staff. These observations are in line with the results from additional models for place of exposure, which showed that contact with a household member and with a colleague were both independently associated with a current or past SARS-CoV-2 infection (household member: adjusted OR 5.97, 95% CI 2.07–17.19; *p* = 0.001. Colleague: adjusted OR 3.33, 95% CI 1.36–8.18; *p* = 0.009 Table 3).

**Table 3:** Two additional multivariable logistic regression models for place of exposure

Variable	Additional model I		Additional model II	
	Adjusted OR (95% CI)	*p* value	Adjusted OR (95% CI)	*p* value
Place of reported exposure				
Household member	5.36 (1.95, 14.77)	0.001	5.97 (2.07, 17.19)	0.001
Friends	0.59 (0.09, 3.79)	0.576	0.61 (0.09, 4.15)	0.615
Colleague	3.24 (1.33, 7.90)	0.010	3.33 (1.36, 8.18)	0.009
Patient	0.91 (0.36, 2.28)	0.840	1.02 (0.38, 2.73)	0.971
Other	2.38 (0.10, 55.47)	0.590	2.44 (0.09, 65.87)	0.596
Age, in years	–	–	1.02 (0.98, 1.06)	0.281
Male gender	–	–	1.09 (0.43, 2.75)	0.855

– Excluded from model, *CI* confidence interval, *OR* odds ratio

## Discussion

The main results of our prospective cohort study among employees at the JUH were the following: (1) The prevalence of a past or current SARS-CoV-2 infection detected by serology and/or PCR among hospital staff members of JUH tripled from 3.2% (initial visit [9]) to 10.8% during the total 13 months study period approximately covering the period from the end of the first to end of the third corona wave in Germany (lasting from the 21st calendar week 2020 to the 24th calendar week 2021) [11]. This finding is comparable to the pooled incidence estimate of SARS-CoV-2 cases of about 12% (95% CI 4–29%) among HCWs reported in a recently published systematic review and meta-analysis with no geographical limitation [12]. The detected SARS-CoV-2 infection rate in our study was numerically higher compared to the prevalence in the community of the city of Jena. According to the official site of the Robert Koch Institute [13], the cumulative number of confirmed COVID-19 cases in the city of Jena was 3902 on 26th April 2021 and 4,382 on 22nd June 2021, corresponding to an infection rate of less than 5% of the overall population. However, due to the assessment of seroprevalence and the intense PCR-based HCW screening described, the detection rate at JUH may have been substantially higher compared to the community. (2) Interestingly, we did not identify occupational contact with COVID-19 patients as a risk factor for infection. Although the majority of hospital staff members reported direct COVID-19 exposure to a SARS-CoV-2 positive patient (67.4%), there was no evidence that this variable increased the risk of infection, likely due to a high overall compliance of 92.4% among HCWs to wear PPE. HCWs caring for COVID-19 patients had a numerically lower infection rate compared to administration staff without any patient care (detected SARS-CoV-2 infection rate: 9.2% among high-risk HCWs versus 12.9% among administration staff) and—in line with this observation—patient-related contact to COVID-19 patients was not identified as a risk factor in the multivariable analyses. This finding is contradictory to other studies that found a higher absolute risk of seropositivity for HCWs with exposure to COVID-19 patients [3, 14, 15]. (3) Similarly to the first assessment of this study [9] and other studies [3, 16], close contact to a SARS-CoV-2 positive household member was identified as the main private risk factor for a SARS-CoV-2 infection. Additionally, participants with a detected SARS-CoV-2 infection reported more frequent direct exposure to a SARS-CoV-2 positive colleague and were more likely to be nurses or administration staff than physicians. The increased risk of nurses may at least partly be attributed to their more frequent contact to and longer contact times with COVID-19 patients as compared to physicians. Furthermore, the increased infection rate in nurses and administration staff relative to physicians may be due to the impact of medical education on infectious risk assessment and respective risk behavior, particularly for non-patient-related contacts. Similarly, a recent scoping review that investigated seroprevalence and risk factors of COVID-19 in 9223 HCWs from eleven countries across Africa found that SARS-CoV-2 seropositivity was associated with lower education and working as a nurse/non-clinical HCW [17]. However, we did not detect an increased infection risk in the group of reception staff. On one hand, this professional group had the fewest participants (only 3% of the study population), which could potentially render it too small to be representative. Additionally, reception staff typically have shorter contact times with patients than nurses, are protected behind a glass screen and -most importantly work in much smaller teams than nursing staff on wards, resulting in less contact with different colleagues.

Hospital staff members can serve as reservoirs, vectors or victims of SARS-CoV-2 cross transmission [4]. Not only can they infect the patients they care for, but they can also spread the virus to other HCWs, further reducing the already limited capacity of health services [3]. To reduce nosocomial transmissions, the medical executive board of our hospital implemented several specific measures that affected both patients and hospital staff. Business trips, particularly to foreign countries, and personal participation in congresses were banned, and repeated PCR testing was mandatory for those returning from risk areas after holidays. However, these parameters were not associated with an increased risk of SARS-CoV-2 infection in our study. As colleagues were identified as the most important source for nosocomial transmissions within the hospital, it was recommended to limit coffee breaks or lunch to a small number of colleagues, while maintaining adequate distance and always eating with the same people. When mandatory masking was not feasible due to eating, drinking, or smoking, speaking should be kept to a minimum.

This study has the following limitations: due to the limited number of study visits (two to three per participant within 1 year) and the lack of mandatory PCR testing among hospital staff, the exact time of SARS-CoV-2 infection detected by serology could not be determined in 16 hospital staff members, and the uncertainty is particularly high in 9 asymptomatic cases. Additionally, the infection rates may be underestimated due to waning antibody titers, particularly after oligo- or asymptomatic infections [18, 19].

In conclusion, our results demonstrate that non-patient-related (most-likely non-protected) contacts to SARS-CoV-2 infected household members and colleagues were the main risk factors, while patient-related contacts (direct contact to COVID-19 patients or body fluids) were not associated with an increased infection risk. Therefore, infection prevention and control strategies should focus more on personal contact between hospital staff members (e.g., using break rooms in small and non-mixed groups only, and enforcing strict universal masking in team meetings) and should improve risk awareness outside the hospital. The lowest infection rate among physicians compared to nurses and administration employees suggests that medical education may have an impact on risk behavior also in the non-occupational setting. This finding underscores the importance of universal masking and educative strategies to decrease the infection risk for hospital employees.

## Supplementary Information

Below is the link to the electronic supplementary material.

Supplementary file1 (PDF 133 KB)

## Data Availability

The datasets used and/or analyzed during the current study are available upon reasonable request from the corresponding author.
