# Supplementary material for: Non-patient-related SARS-CoV-2 exposure from colleagues and household members poses the highest infection risk for hospital employees in a German university hospital: follow-up of the prospective Co-HCW seroprevalence study
Source: Infection. 2023 Feb 15;51(4):1051–9. doi: 10.1007/s15010-023-01995-z (PMC9928590; doi:10.1007/s15010-023-01995-z)
Supplement: Supplementary file 1 — Supplementary file1 (PDF 133 KB) [file 15010_2023_1995_MOESM1_ESM.pdf]

Table S1. Complete multivariable regression models assessing potential risk factors for a SARS-CoV-2 infection (detected by serology and/or PCR) among hospital staff members

Variable	adjusted OR (95% CI)	p-value
Age, in years	1.00 (0.97, 1.03)	0.962
Male gender	1.18 (0.57, 2.43)	0.633
Profession*[1]		
Physician	ref.	0.108
Nurse	5.57 (1.24, 25.12)	0.025
Reception staff	3.05 (0.25, 37.65)	0.384
Administration staff	4.92 (1.07, 22.64)	0.041
Age, in years	1.00 (0.96, 1.03)	0.835
Male gender	1.13 (0.50, 2.54)	0.771
COVID-19 risk group according to working place		
High-risk	ref.	0.644
Intermediate-risk	1.15 (0.46, 2.89)	0.763
Low-risk	1.52 (0.58, 3.98)	0.397
Age, in years	1.00 (0.97, 1.03)	0.826
Male gender	1.17 (0.56, 2.44)	0.676
Reported COVID-19 exposure	7.19 (2.86, 18.11)	<0.001
Age, in years	1.03 (0.99, 1.06)	0.122
Male gender	1.12 (0.53, 2.36)	0.766
Place of reported exposure: household member*[3]	4.46 (2.06, 9.65)	<0.001
Age, in years	1.02 (0.99, 1.06)	0.212
Male gender	1.02 (0.43, 2.39)	0.967
Place of reported exposure: friends*[3]	0.52 (0.11, 2.35)	0.394
Age, in years	1.02 (0.98, 1.06)	0.309
Male gender	1.04 (0.45, 2.38)	0.925
Place of reported exposure: colleague*[3]	2.30 (1.10, 4.79)	0.026
Age, in years	1.02 (0.98, 1.05)	0.402
Male gender	1.07 (0.47, 2.48)	0.867
Place of reported exposure: patient*[3]	0.36 (0.18, 0.75)	0.007
Age, in years	1.01 (0.98, 1.05)	0.528
Male gender	1.15 (0.50. 2.68)	0.742
Place of reported exposure: other*[3]	2.60 (0.22, 30.41)	0.446
Age, in years	1.02 (0.98, 1.06)	0.304
Male gender	0.95 (0.41, 2.20)	0.913
Accident with biological material	2.77 (0.54, 14.23)	0.222
Age, in years	1.00 (0.97, 1.03)	0.945
Male gender	1.15 (0.55, 2.38)	0.711
Compliance to wear PPE*[2]	0.58 (0.11, 2.94)	0.507
Age, in years	0.99 (0.94, 1.05)	0.855
Male gender	0.96 (0.31, 2.94)	0.945
Use of public transport	1.77 (0.64, 4.54)	0.235
Age, in years	1.00 (0.97, 1.03)	0.987
Male gender	1.20 (0.58, 2.48)	0.625
Number of household members	0.99 (0.77, 1.27)	0.918
Age, in years	1.00 (0.97, 1.03)	0.965
Male gender	1.18 (0.57, 2.43)	0.661
More than 1 household member	0.92 (0.44, 1.95)	0.835
Age, in years	1.00 (0.97, 1.03)	0.956
Male gender	1.17 (0.57, 2.42)	0.668
Returning from risk area	1.25 (0.59, 2.65)	0.562

Age, in years	1.00 (0.97, 1.03)	0.942
Male gender	1.17 (0.57, 2.43)	0.664
Travel to abroad	1.20 (0.59, 2.44)	0.614
Age, in years	1.00 (0.97, 1.03)	0.930
Male gender	1.19 (0.57, 2.46)	0.641
Participation at events with ≥5 persons	1.32 (0.70, 2.51)	0.389
Age, in years	1.00 (0.97, 1.03)	0.832
Male gender	1.17 (0.56, 2.42)	0.675

[*1] 71 persons with "other profession" were excluded from analysis
[*2] Information is missing for 262 participants
[*3] Among participants with reported place of exposure
Abbreviations: CI, confidence interval; OR, odds ratio; PCR, polymerase chain reaction; PPE, personal protective equipment; ref., reference.

Table S2. Potential risk factors for a current or past SARS-CoV-2 infection (detected by serology and/or PCR) among male hospital staff

Variable	Male gender		
	Overall (N=91)	Detected Infection	
		Yes (N=11)	No (N=80)
Age, in years	41.0 (33.0, 48.0)	46.0 (40.0, 50.5)	40.5 (32.8, 48.0)
Male gender	91 (100.0%)	11 (100.0%)	80 (100%)
Profession			
Physician	21 (23.1%)	0 (0.0%)	21 (26.2%)
Nurse	21 (23.1%)	5 (45.5%)	16 (20.0%)
Reception staff	3 (3.3%)	1 (9.1%)	2 (2.5%)
Administration staff	34 (37.4%)	3 (27.3%)	31 (38.8%)
Other profession	12 (13.2%)	2 (18.2%)	10 (12.5%)
COVID-19 risk group according to working place			
High-risk	25 (27.5%)	3 (27.3%)	22 (27.5%)
Intermediate-risk	32 (35.2%)	5 (45.5%)	27 (33.8%)
Low-risk	34 (37.4%)	3 (27.3%)	31 (38.8%)
Reported COVID-19 exposure	54 (59.3%)	9 (81.8%)	45 (56.2%)
among them: Place of reported exposure			
Household member	10 (18.5%)	1 (11.1%)	9 (20.0%)
Friend	8 (14.8%)	1 (11.1%)	7 (15.6%)
Colleague	11 (20.4%)	4 (44.4%)	7 (15.6%)
Patient	42 (77.8%)	6 (66.7%)	36 (80.0%)
Other	2 (3.7%)	1 (11.1%)	1 (2.2%)
Accident with biological material	3 (3.3%)	0 (0.0%)	3 (3.8%)
Compliance to wear PPE[1]	42 (95.5%)	4 (80.0%)	38 (97.4%)
Use of public transport	6 (6.6%)	0 (0.0%)	6 (7.5%)
Household size			
Number of members	3.0 (2.0, 4.0)	3.0 (2.0, 4.0)	3.0 (1.0, 4.0)
>1 member	69 (75.8%)	10 (90.9%)	59 (73.8%)
Returning from risk area	18 (19.8%)	3 (27.3%)	15 (18.8%)
Travel to abroad	18 (19.8%)	2 (18.2%)	16 (20.0%)
Participation at an event with ≥ 5 persons	46 (50.5%)	6 (54.5%)	40 (50.0%)

[1] Information is missing from 47 participants

Table S3. Potential risk factors for a current or past SARS-CoV-2 infection (detected by serology and/or PCR) among female hospital staff

Variable	Female gender		
	Overall (N=315)	Detected Infection	
		Yes (N=33)	No (N=282)
Age, in years	42.0 (34.0, 51.0)	41.0 (31.0, 51.0)	42.0 (34.2, 50.8)
Male gender	0 (0.0%)	0 (0.0%)	0 (0.0%)
Profession			
Physician	45 (14.3%)	2 (6.1%)	43 (15.2%)
Nurse	104 (33.0%)	13 (39.4%)	91 (32.3%)
Reception staff	9 (2.9%)	0 (0.0%)	9 (3.2%)
Administration staff	98 (31.1%)	14 (42.4%)	84 (29.8%)
Other profession	59 (18.7%)	4 (12.1%)	55 (19.5%)
COVID-19 risk group according to working place			
High-risk	51 (16.2%)	4 (12.1%)	47 (16.7%)
Intermediate-risk	166 (52.7%)	15 (45.5%)	151 (53.5%)
Low-risk	98 (31.1%)	14 (42.4%)	84 (29.8%)
Reported COVID-19 exposure	170 (54.0%)	29 (87.9%)	141 (50.0%)
among them: Place of reported exposure			
Household member	33 (19.4%)	15 (45.5%)	18 (12.8%)
Friend	12 (7.1%)	1 (3.4%)	11 (7.8%)
Colleague	49 (28.8%)	12 (41.4%)	37 (26.2%)
Patient	109 (64.1%)	12 (41.4%)	97 (68.8%)
Other	1 (0.6%)	0 (0.0%)	1 (0.7%)
Accident with biological material	5 (1.6%)	2 (6.1%)	3 (1.1%)
Compliance to wear PPE[1]	91 (91.0%)	11 (91.7%)	80 (90.9%)
Use of public transport	30 (9.5%)	6 (18.2%)	24 (8.5%)
Household size			
Number of members	2.0 (2.0, 4.0)	2.0 (1.0, 3.0)	2.0 (2.0, 4.0)
>1 member	250 (79.4%)	24 (72.7%)	226 (80.1%)
Returning from risk area	61 (19.4%)	7 (21.2%)	54 (19.1%)
Travel to abroad	81 (25.7%)	10 (30.3%)	71 (25.2%)
Participation at an event with ≥ 5 persons	151 (47.9%)	18 (54.5%)	133 (47.2.0%)

[1] Information is missing from 215 participants
